# A Rare Case of Noninsulinoma Pancreatogenous Hypoglycemia Syndrome

**DOI:** 10.1155/2012/164305

**Published:** 2012-11-20

**Authors:** Jeffrey Nadelson, Alan Epstein

**Affiliations:** Section of Gastroenterology, Department of Medicine, Roger Williams Medical Center, Boston University School of Medicine, 825 Chalkstone Avenue, Providence, RI 02908, USA

## Abstract

As the obesity pandemic continues to worsen and medical interventions remain only moderately effective, bariatric surgery remains an important option for patients. In certain instances, patients who have undergone the procedure experience postprandial symptoms of neuroglycopenia caused by noninsulinoma pancreatogenous hypoglycemia syndrome (NIPHS). NIPHS is a recently described syndrome and is also very rare, and therapeutic approaches are still under debate. We report the case of a 51-year-old female who underwent Roux-en-Y gastric bypass and presented with episodic postprandial hypoglycemia 2 years after surgery. An insulinoma was absent from all abdominal imaging. Fasting C-peptide, insulin, and glucose were normal. Due to the possibility of NIPHS, clinical treatment was commenced with acarbose, leading to a significant reduction of hypoglycemic episodes. NIPHS occurs in approximately 0.5% to 7% of patients with hyperinsulinemic hypoglycemia. Sporadic hypoglycemia postgastric bypass is an important entity that should be understood by all surgeons and internists who are involved in postgastric bypass care.

## 1. Background

Gastric bypass surgery for patients with severe obesity is increasing due to the obesity epidemic in the United States [1]. The number of bariatric surgeries in the United States has increased by 16% from the early 1990s to 2003 [2]. As the obesity pandemic continues to worsen and medical interventions remain only moderately effective, bariatric surgery remains an important option for patients. In certain instances, patients who have undergone the procedure experience postprandial symptoms of neuroglycopenia, dumping syndrome [3], insulinoma [4], and noninsulinoma nesidioblastosis [5] or also known as noninsulinoma pancreatogenous hypoglycemia syndrome (NIPHS). Dumping syndrome alone consists of diaphoresis, weakness, dizziness, and flushing, but not neuroglycopenia [3]. NIPHS postprandial symptoms of neuroglycopenia include cognitive impairment, behavioral changes, confusion, depression, diaphoresis, weakness, dizziness, and, at lower plasma glucose concentrations, generalized or focal seizures and coma. The pathogenesis of NIPHS in patients who have undergone Roux-en-Y gastric bypass is still unclear; however, it is thought to be due to hypersecretion of insulin from beta-cell hyperplasia [6] and increased periductular islets [7].

We report herein a very rare case of postprandial symptoms of neuroglycopenia as a result of NIPHS in a woman who has undergone Roux-en-Y gastric bypass with multiple revisions and gastrectomy.

## 2. Clinical Presentation and Clinical Course

A 51-year-old female with a past medical history of Roux-en-Y gastric bypass with multiple revisions and gastrectomy secondary to multiple gastric fistulae, obesity, asthma, and hypertension was admitted to a university medical center for multiple episodes of hypoglycemic symptoms postgastric bypass revision. During these episodes of hypoglycemia, the patient showed abrupt symptoms of palpitations, confusion, weakness, diaphoresis, shaking, trembling, tingling and numbness in her extremities, and temporary paralysis. The laboratory data on admission revealed a glucose level of 35 mg/dL. She stated that the neuroglycopenic symptoms would occur 4-5 times per week, since the gastric bypass revision was completed 3 years ago, however, did not seek medical attention. Her hypoglycemic symptoms would occur 3-4 hours after consumption of a meal, and during these neuroglycopenic episodes, she would check her blood sugar and the levels ranged between 10–25 mg/dL. The symptoms would be relieved by carbohydrate dense foods. During the admission, patient was kept n.p.o except for p.o. fluids. Blood sugar finger sticks were checked hourly, and the plan was to draw proinsulin, insulin, C-peptide, cortisol, and beta-hydroxybutyrate levels once her blood sugar decreased to less than 55 mg/dL with symptoms or less than 45 mg/dL without symptoms. On the third day of fasting, the patient had an episode of neuroglycopenia with a blood sugar of 32 mg/dL. Laboratory findings revealed a proinsulin level of 17, insulin 3.3, C-peptide 759, cortisol 8.2, and beta-hydroxybutyrate level 0.1. Endocrine examinations to exclude other causes of hypoglycemia, such as hypopituitarism and adrenal insufficiency, were within the normal range. With the history of Roux-en-Y gastric bypass, hypoglycemia and the previously described symptoms were thought to be consistent with insulin-producing lesions including an insulinoma; however, imaging studies failed to detect evidence of pancreatic masses or insulinoma. Patient underwent an abdominal CT as well as an OctreoScan study. Although imaging studies showed no mass, the patient was offered the Whipple procedure as a possible treatment; however, the patient declined. The neuroglycopenic symptoms described previously were resolved, and the patient was discharged home with a diagnosis of postprandial hypoglycemia. 

The patient did not experience any episodes of hypoglycemia for the next three years after her initial presentation and discharge until she experienced an episode of altered mental status in the early morning and was brought to a local community hospital by family members. While in the emergency department (ED), the patient stated that she awoke spontaneously, found herself lying on the floor, and does not recall how she arrived there. While lying on the floor of her bedroom, she was unable to move any of her extremities for approximately twenty minutes. She stated that she experienced the same neuroglycopenic symptoms that were described previously 3 years prior. The urine toxicology screen in the ED showed no evidence of alcohol or drug abuse. Laboratory findings revealed a low fasting blood sugar of 72 mg/dL. The neuroglycopenic symptoms were relieved after ingestion of juice. Patient was discharged home with a diagnosis of hypoglycemia with no discharge medications or followup scheduled. 

Patient continued to have neuroglycopenic episodes and; therefore, presented to her primary care physician, since no etiology of these episodes had been found. After meeting with the primary care physician, patient underwent another abdominal CT scan that once again showed no evidence of pancreatic masses or insulinomas. Patient was placed on acarbose 25 mg twice a day by mouth and advised to take it with meals. A cardiology, neurology, and endocrinology evaluations were sought to find the source of the syncopal episodes. Patient underwent a transthoracic echocardiogram (TTE), which revealed an estimated EF of 60%, no aortic stenosis, no cardiomyopathies, and pulmonary artery systolic pressure to be less than 30 mmHg. Electrocardiogram was normal. Cardiac causes of syncope were ruled out. Electroencephalography was normal, and no neurologic source was found to cause her syncope. 

 Due to the patient's history of gastric bypass, NIPHS was high on the differential, and acarbose was increased from 50 mg twice to three times a day. The patient went symptom free from the time of the first visit to the follow-up appointment 3 months later. At the second visit, a continual glucose monitor was placed underneath the skin in her abdomen. Blood sugar measurements were taken every five minutes for 5 days. During these 5 days of recording, the patient kept a journal and recorded the times of her meals and acarbose administration. Morning readings averaged 35 mg/dL, and afternoon readings averaged 40 mg/dL. After examining the blood sugar measurements over the five-day period, it was recommended that she has to increase the dose of acarbose to 150 mg three times a day by mouth and to refrain from foods that are high in carbohydrates and sugar. She is currently very compliant with her medications and her diet and has continued to be symptom free for 1 year. 

## 3. Discussion

NIPHS is very rare and occurs in approximately 0.5% to 7% of patients with hyperinsulinemic hypoglycemia [8–10]. NIPHS is characterized by endogenous hyperinsulinemic hypoglycemia that is not caused by insulinoma. Pancreatic specimens from patients with this syndrome typically show beta-cell hypertrophy, islets with enlarged and hyperchromatic nuclei, and increased periductular islets [5, 7, 11]. Nesidioblastosis has mainly been described in neonates [12]; however, there is emerging recognition that postprandial hypoglycemia in adults after bypass may be due to endogenous hyperinsulinemia from abnormal islets, as a result of either nesidioblastosis or insulinoma. 

The patient presented here has had frequent hypoglycemic episodes mostly in the late evening and early mornings, independent of food consumption. Sporadic hyperinsulinemic hypoglycemia is the main clinical feature of nesidioblastosis; however, gastrectomies have been shown to cause episodes of hypoglycemia as well. This patient has had a gastrectomy due to multiple gastric fistulae, in addition to multiple revisions to her gastric bypass. Marks and Rose state that gastrectomies may evoke hypoglycemia, which is sometimes severe enough to cause loss of consciousness [13]. Another interesting finding is that postprandial hypoglycemia following a gastrectomy usually occurs 1.5 to 3 hours after food ingestion [14]. This patient's neuroglycopenia symptoms occurred at night and early morning, therefore, suggesting that the neuroglycopenic episodes were most likely due to nesidioblastosis rather than the gastrectomy. 

 It is possible that the nesidioblastosis was a consequence of gastric bypass. Service et al. studied six patients from 2000 to 2004, who had undergone Roux-en-Y gastric bypass for extreme obesity and were victims of repeated episodes of postprandial hypoglycemia. All of the six patients they studied during that time period who had undergone gastric bypass were found to have nesidioblastosis. During the two years of the study, nine patients at Massachusetts General Hospital had nesidioblastosis without a history of gastric bypass. Therefore, 40% of patients who had nesidioblastosis had undergone gastric bypass [6]. They discovered that the frequency of nesidioblastosis in gastric bypass patients far exceeded that in the general population. 

 In summary, we report here a rare case of NIPHS in a patient with a history of Roux-en-Y gastric bypass. Sporadic hypoglycemia postgastric bypass is an important entity that should be understood by all surgeons and internists who are involved in postgastric bypass care. The optimal treatment for this condition requires further study. 
